# Phytochemical Composition and Antipseudomonal Activity of *Allanblackia gabonensis* (Clusiaceae) Extracts Alone and With Antibiotics Against Drug‐Resistant Clinical Isolates

**DOI:** 10.1155/sci5/6070077

**Published:** 2026-03-02

**Authors:** Céline Brinda Sonfack, Aimé Gabriel Fankam, Brenda Ngueffo Tiwa, Michael Francis Kengne, Armelle Tsafack Mbaveng, Victor Kuete

**Affiliations:** ^1^ Department of Biochemistry, University of Dschang, Dschang, Cameroon, univ-dschang.org

**Keywords:** *Allanblackia gabonensis*, aminoglycosides, antibiotic resistance modulation, multidrug-resistant, *Pseudomonas aeruginosa*, synergy

## Abstract

**Background:**

The discovery of alternative therapies for illnesses due to multidrug‐resistant (MDR) bacteria is emerging as a global health crisis. This study aimed to assess the antibacterial efficacy of *Allanblackia gabonensis* extracts, both alone and in conjunction with antibiotics, against MDR *Pseudomonas aeruginosa*.

**Methods:**

The extracts were subjected to phytochemical analysis using both qualitative and quantitative standard tests. The microdilution assay was used to evaluate the antibacterial properties and antibiotic resistance‐modifying potential of the extracts. The interaction effect between the antibiotics and extracts was determined by checkerboard assay. Catalase activity and lipid peroxidation were assessed by measuring the foam height and malondialdehyde concentration, respectively.

**Results:**

The extracts exhibited minimum inhibitory concentrations (MICs) ranging from 16 to 2048 μg/mL. The hexane extracts of the leaves (HLE) and bark (HBE) displayed the highest antibacterial activity, with MIC ≤ 32  μg/mL against at least two investigated isolates. Furthermore, HLE decreased catalase activity and increased lipid peroxidation in *P. aeruginosa* D130. The extracts at their sub‐inhibitory concentration (MIC/8) enhanced the activity of antibiotics, mainly aminoglycosides (amikacin, streptomycin, and gentamicin), by 2‐ to 256‐fold against selected MDR *P. aeruginosa*. Among these extracts, the hexane and methanol bark extracts exhibited synergy (∑FIC ≤ 0.5)) in combination with these antibiotics. All examined extracts contained alkaloids, phenols, and triterpenes. Moreover, dichloromethane/methanol and methanol leaf extracts presented the highest phenolic content.

**Conclusion:**

Overall, the leaf and bark hexane extracts of *A. gabonensis* could serve as candidates for the discovery of new antibiotics, while its bark extracts might be used in conjunction with antibiotics to manage infections involving multidrug‐resistant *P. aeruginosa*.

## 1. Introduction

After many decades of using antibiotics to treat bacterial infections in humans and animals, bacteria have developed alarming levels of resistance and multiple resistance to many antibiotics. Approximately 2.8 million infections and 35,000 deaths are associated with antibiotic resistance annually [1]. Among them, *Pseudomonas aeruginosa* is a multidrug‐resistant (MDR) and opportunistic bacterium that is a leading cause of mortality and morbidity in patients with cystic fibrosis and immunocompromised individuals [2, 3]. In 2017, MDR *P. aeruginosa* was considered a serious threat by the World Health Organization and Centers for Disease Control and Prevention (CDC), accounting for at least 32,600 cases, 2700 deaths, and US $767 million in attributable healthcare costs annually [4, 5]. With high‐level intrinsic resistance, *P. aeruginosa* counters antibiotic action through various resistance mechanisms, such as low outer membrane permeability, enzyme inactivation, and the expression of active efflux pumps [2, 6, 7]. Due to its remarkable capacity to resist antibiotics, the eradication of *P. aeruginosa* has become increasingly difficult. Consequently, the development of novel therapeutic strategies to treat infections caused by MDR *P. aeruginosa* is becoming necessary. Given this, medicinal plants known for their antimicrobial properties constitute a potential reservoir for the discovery of new substances effective against multiresistant antibiotic phenotypes [8, 9]. Moreover, the natural product combination may enhance the interaction of antimicrobial agents with their targets within the pathogen, thereby reducing the development of resistance. This approach can mitigate toxicity by utilizing lower amounts of both drugs [10, 11]. Numerous studies have demonstrated the antibacterial potential of several medicinal plant extracts and their constituents, and even the ability of some of them to improve the effectiveness of conventional antibiotics [12, 17]. *Allanblackia gabonensis* is a plant belonging to the Clusiaceae family and is largely distributed in the rainforests of Africa, mainly in Eastern Nigeria, Southern and Northern Angola, Uganda, and Cameroon [18, 19]. It is used in traditional medicine to relieve pain and rheumatism, improve virility in men, and treat dysentery, colds, and toothaches [20, 21]. Many phytochemical classes, including xanthones, benzophenones, flavonoids, and phytosterols, have been isolated from *A. gabonensis* stem bark and fruit extracts [22, 23]. Moreover, some previous studies have reported the antileishmanial [21], antiplasmodial [22], antimicrobial [21, 23–25], analgesic, anti‐inflammatory [26], hepato‐nephroprotective, antioxidant [27], and anticancer [28, 29] properties of extracts and isolated compounds from this plant. This study aimed to assess the antibacterial efficacy of *A. gabonensis* extracts, both alone and in conjunction with antibiotics, against MDR *Pseudomonas aeruginosa*. Additionally, the preliminary phytochemical composition of the extracts and the effect of the most active extract on the antioxidant system of *P. aeruginosa* were determined.

## 2. Materials and Methods

### 2.1. Plant Material

Fresh leaves and bark of *Allanblackia gabonensis* (Clusiaceae) were collected from a field in November 2023 at Dschang, West Cameroon. The plant sample was botanically identified at the National Herbarium of Cameroon, Yaoundé, where the voucher has been deposited under the registration number 17275 SRF/Cam.

### 2.2. Preparation of Plant Extracts

Each plant organ was washed in clean water, cut into small pieces, and dried at room temperature in the shade. The dried material was ground, and 200 g of powder was extracted by maceration in 600 mL of hexane, the mixture of dichloromethane/methanol (CH_2_Cl_2_/MeOH, 1:1), and methanol (MeOH) for 48 h at room temperature. Each crude extract was filtered on Whatman paper No. 1 and concentrated under reduced pressure using a rotary evaporator (BÜCHI R‐200) at 40°C. The residual solvent was removed by drying the extracts at 40°C in an oven. The hexane, CH_2_Cl_2_/MeOH, and MeOH extract yields were 1.39%, 4.57%, and 9.94%, respectively, for the leaves, and 1.88%, 11.41%, and 16.07% for the bark.

### 2.3. Preliminary Phytochemical Screening

#### 2.3.1. Qualitative Phytochemical Screening

To identify the main classes of secondary metabolites likely to be responsible for the biological activities of *A. gabonensis* extracts, a qualitative phytochemical screening was carried out. The standard methods, such as Mayer’s test (detection of alkaloids), Salkowski’s test (detection of terpenoids), the froth test (detection of saponins), the ferric chloride test (detection of phenols), the lead acetate test (detection of flavonoids), the ferric chloride test (detection of tannins), and the HCl test (detection of anthocyanins), were used [30, 31].

#### 2.3.2. Determination of the Total Phenolic Content (TPC)

The TPC of *A. gabonensis* extracts was assessed using the Folin–Ciocalteu method as previously described [16, 32]. The reaction mixture comprised 0.02 mL of extract (2 mg/mL), 1 mL of tenfold diluted Folin–Ciocalteu reagent, and 0.8 mL of 20% sodium carbonate solution. The mixture was stirred and incubated at 37°C for 30 min, and its absorbance was quantified using a spectrophotometer (Biobase BK‐D590) at 765 nm. Results were expressed as milligrams of gallic acid equivalents per gram of extract (mg GAE/g) utilizing a calibration curve. Each sample underwent three assays.

### 2.4. Test Organisms and Culture Conditions

The antibacterial activity of extracts was evaluated on nineteen *P. aeruginosa* strains, including one strain (ATCC27853) from the American Type Culture Collection and 18 MDR clinical isolates (B026, B178, D100, D130, K033, K126, K130, K139, K142, K261, K272, K290, K294, T076, T106, T138, T201, and PA124). Their resistance profiles are presented in the supporting information (Table S1, supporting information). These bacteria were cultured and maintained at 37°C for 18 h in Petri dishes containing Mueller–Hinton agar (MHA, ReadyMED, USA). Mueller–Hinton broth (MHB, ReadyMED, USA) was used for the preparation of the bacterial inoculum and to carry out antibacterial assays.

### 2.5. Inoculum Preparation

About 4–6 colonies were taken from a pure culture of the test bacteria and mixed with sterile distilled water to have a bacterial suspension. The absorbance of the suspension was then measured at 600 nm using a spectrophotometer (Biobase BK‐D590) with a 1‐cm light path until the absorbance reached 0.08–0.1, equivalent to 1.5 × 10^8^ CFU/mL of bacteria. Thereafter, the obtained suspension was diluted with MHB in 10‐mL sterile test tubes to obtain a concentration of 2 × 10^6^ CFU/mL.

### 2.6. Antibacterial Activity of Plant Extracts

#### 2.6.1. Determination of Minimum Inhibitory Concentration (MIC)

The MIC of the extracts was determined using the iodonitrotetrazolium chloride (INT) assay as previously described [33, 34]. Preparation of test sample solutions was done by dissolving the extract in dimethyl‐sulfoxide (DMSO, BDH Chemicals Ltd, Poole, England)/MHB (10:90, v/v) to yield stock solutions of 4096 μg/mL for extracts and 1024 μg/mL for streptomycin used as a reference antibacterial drug. A volume of 100 μL of the resulting solutions was serially diluted twice in a 96‐well microplate containing 100 μL of MHB. Thereafter, 100 μL of inoculum (10^6^ CFU/mL) was added to each well, followed by incubation at 37°C for 18 h. Thereafter, 40 μL of INT solution (0.2 mg/mL) was added to each well, and the microplate was incubated at 37°C for 30 min. MIC was defined as the lowest concentration of the sample that completely prevented bacterial growth (absence of pink coloration in the wells). The final concentrations of extracts and antibiotics varied from 16 to 2048 μg/mL and from 2 to 256 μg/mL, respectively. Wells containing only MHB were used as a negative control, while those containing bacterial inoculum and DMSO served as a growth control. The maximum final concentration of DMSO was 2.5% and did not affect the bacterial growth. Each experiment was carried out in duplicate and repeated three times. The activity of the extracts against *P*. *aeruginosa* was defined as follows: outstanding activity (MIC ≤ 32 μg/mL), excellent activity (32 < MIC ≤ 128 μg/mL), very good activity (128 < MIC ≤ 256 μg/mL), good activity (256 < MIC ≤ 512 μg/mL), average activity (512 < MIC ≤ 1024 μg/mL), weak activity, or not active (MIC > 1024 μg/mL) [35].

#### 2.6.2. Determination of Minimum Bactericidal Concentrations (MBCs)

The MBC of each sample was assessed by subculturing 50 μL of the suspension from the wells that showed no growth after the MIC test in a sterile 96‐well microplate containing 150 μL of fresh MHB, followed by a re‐incubation at 37°C for 48 h. Thereafter, 40 μL of INT solution (0.2 mg/mL) was added as described above. The lowest sample concentration that killed the bacteria (absence of pink coloration in the wells) was identified as MBC [34]. An extract was considered to have a bactericidal effect if MBC/MIC ≤ 4 and a bacteriostatic effect if MBC/MIC > 4 [36].

### 2.7. Evaluation of Modes of Action of the Active Extract

#### 2.7.1. Catalase Assay

The effect of the hexane leaf extract (HLE) on the catalase activity of *P. aeruginosa* D130 was carried out as previously described [17, 37]. Briefly, fresh colonies of *P. aeruginosa* D130 were cultured overnight in 4 mL of MHB containing 1 mL of HLE (MIC and 2xMIC) or without extract (untreated cells or control). Ciprofloxacin (MIC) was used as the reference antibacterial drug. 100 μL of the appropriate test samples was transferred into a test tube, followed by the addition of 100 μL of 1% Triton X‐100 (HiMedia, Mumbai, India) and 100 μL of 30% (v/v) H_2_O_2_. A tube containing distilled water, Triton X‐100%, and 30% (v/v) H_2_O_2_ was used as a blank control. The catalase inhibition effect was based on the height of foam (cm) of the test tubes compared to that of the control (untreated cells).

#### 2.7.2. Lipid Peroxidation Assay

The lipid peroxidation potential of HLE was assessed by measuring the concentration of malondialdehyde (MDA) [37, 38]. Briefly, fresh colonies of *P. aeruginosa* D130 were cultured overnight in 4 mL of MHB containing 1 mL of HLE (MIC and 2xMIC) or polymyxin B (MIC) used as the reference antibacterial drug. Thereafter, 1 mL of the test culture, 1 mL of thiobarbituric acid, and 1 mL of trichloroacetic acid were introduced into a screw‐cap test tube, mixed well, and heated for 10 min at 100°C in a water bath. After the test tubes were allowed to cool, they were centrifuged for 20 min at 5000 rpm. The supernatants were then collected, and their absorbances were measured at 535 nm against the control. A tube containing 1 mL of water instead of the test sample was used as the control (untreated cells), whereas a tube containing 1 mL of water instead of bacterial suspension was used as the blank tube. The MDA concentration (μM) was calculated based on its millimolar absorbance coefficient (*E*_0_ = 156 cm^−1^·mM^−1^) using the following equation:

(1) MDAµM=sample absorbance−control absorbanceE0×103.

### 2.8. Evaluation of the Enhancement of Antibiotic Activity

#### 2.8.1. Analysis of the Antibiotic Resistance Modulation Effect of *A. gabonensis* Extracts

The antibiotic‐modifying activity of the extract was carried out as previously described [12, 39]. The MIC of ciprofloxacin, levofloxacin, streptomycin, gentamicin, amikacin, cefixime, and cefotaxime (Sigma‐Aldrich) was assessed with or without the extracts at their subinhibitory concentration (MIC/8). The modulation factor, defined as the ratio of the MIC of the antibiotic alone to that of the antibiotic in the presence of the extract, was calculated. A modulation factor ≥ 2 has been set as the threshold for biological significance of antibiotic resistance modulation effect [40]. Each assay was performed in duplicate and repeated three times.

#### 2.8.2. Evaluation of the Interaction Effects Between Antibiotics and *A. gabonensis* Extracts

The interactions between extracts and aminoglycosides (amikacin, gentamicin, and streptomycin) were investigated using the checkerboard method as previously described [17, 41]. Briefly, the antibiotic was twofold serially diluted in a sterile 96‐well microplate, from Row A to Row G, followed by the dilution of the extract from column 1 to 10. Thereafter, 100 μL of inoculum was added to all wells except the last four wells of Column 12 and the last well of Column 11, which were used as controls. The microplates were then incubated for 18 h at 37°C, and the MIC was determined as previously described. The fractional inhibitory concentration index (∑FIC) for all the combinations was determined using the following formula:

(2) ∑FIC= MIC of extract in combinationMIC of extract alone+ MIC of antibiotic in combinationMIC of antibiotic alone.

The interaction was considered as follows: synergy (∑FIC ≤ 0.5); additivity (0.5 < ∑FIC ≤ 1); indifferent if (∑FIC > 1–2); and antagonism (∑FIC > 2) [42].

### 2.9. Data Analysis

In this study, each experiment was done in triplicate, and data were reported as mean ± standard deviation (mean ± SD). One‐way analysis of variance followed by Tukey’s post hoc multiple comparison test at a significance level of *p* < 0.05 was employed to compare the means. GraphPad Prism software for Windows, Version 8.4.2, was used for data analysis.

## 3. Results

### 3.1. Phytochemical Composition of Extracts

#### 3.1.1. Qualitative Phytochemical Composition

The results of the phytochemical screening showed that all the screened phytochemical classes were present in the dichloromethane/methanol leaf extract (DMLE), methanol leaf extract (MLE), and methanol bark extract (MBE). Alkaloids, phenols, and triterpenes were detected in all the extracts, whereas flavonoids, tannins, saponins, anthocyanins, and anthraquinones were selectively present in these extracts (Table 1).

**TABLE 1:** Qualitative chemical composition of *A. gabonensis* extracts.

Phytochemical classes	Extracts					
	HLE	DMLE	MLE	HBE	DMBE	MBE
Alkaloids	+	+	+	+	+	+
Phenols	+	+	+	+	+	+
Flavonoids	—	+	+	—	+	+
Tannins	—	+	+	—	+	+
Triterpenes	+	+	+	+	+	+
Saponins	+	+	+	—	+	+
Anthocyanins	—	+	+	—	—	+
Anthraquinones	—	+	+	+	—	+

*Note:* (+): present; (—): absent.

Abbreviations: DMBE, dichloromethane/methanol bark extract; DMLE, dichloromethane/methanol leaf extract; HBE, hexane bark extract; HLE, hexane leaf extract; MBE, methanol bark extract; MLE, methanol leaf extract.

#### 3.1.2. TPC of Extracts

**FIGURE 1:**
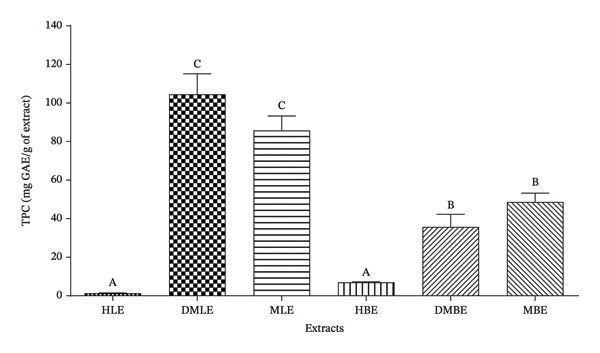
Total phenolic content of *A. gabonensis* extracts. TPC: total phenolic content; GAE: gallic acid equivalents. HLE: hexane leaf extract; DMLE: dichloromethane/methanol leaf extract; MLE: methanol leaf extract; HBE: hexane bark extract; DMBE: dichloromethane/methanol bark extract; MBE: methanol bark extract.

The results showed that the DMLE had a significantly higher TPC than the other extracts (104.50 ± 10.61 mg GAE/g of extract). It was followed by the MLE, MBE, dichloromethane/methanol bark extract (DMBE), hexane bark extract (HBE), and HLE, with values of 85.65 ± 7.56 mg GAE/g, 48.40 ± 2.40 mg GAE/g, 35.53 ± 3.16 mg GAE/g, 7.00 ± 0.23 mg GAE/g, and 0.88 ± 0.01 mg GAE/g, respectively (Figure 1).

### 3.2. Antibacterial Activity of *A. gabonensis* Extracts

The antibacterial activity of the different crude extracts was determined through the determination of their MICs and MBCs against *P. aeruginosa*. The results show that the tested extracts have variable antibacterial activities with MICs ranging from 16 to 2048 μg/mL. The HBE of *A. gabonensis* was active against 94.73% (18/19), followed by the DMBE (78.94%), the DMLE and MLE, and the HBE and MBE (73.98%). Overall, HLE, HBE, MLE, and MBE extracts showed high activities (MIC ≤ 128 μg/mL) on at least one of the tested *P. aeruginosa* isolates. HLE and MBE showed outstanding activity (MIC ≤ 32 μg/mL) against *P. aeruginosa* D130, K290, and K294, while HBE was active against *P. aeruginosa* K142 and T076. Furthermore, HBE, MLE, and MBE showed excellent activity (32 < MIC ≤ 128 μg/mL) against *P. aeruginosa* K294, K290, and T076, respectively. The reference antibiotic, streptomycin, presented MICs ranging from 16 to 128 μg/mL. In most cases, MBCs were not detected up to 2048 μg/mL for the extracts and 128 μg/mL for streptomycin (Table 2).

**TABLE 2:** MIC and MBC of *A. gabonensis* extracts against *Pseudomonas aeruginosa*.

*P. aeruginosa*	HLE		DMLE		MLE		HBE		DMBE		MBE		Streptomycin	
	MIC	MBC	MIC	MBC	MIC	MBC	MIC	MBC	MIC	MBC	MIC	MBC	MIC	MBC
ATCC27853	512	—	2048	—	—	—	256	—	1024	—	2048	—	128	128
B026	1024	2048	1024	—	512	—	2048	—	—	—	2048	—	128	> 128
B178	2048	—	—	—	—	—	1024	—	2048	—	—	—	> 128	> 128
D100	—	—	256	2048	—	—	2048	—	—	—	2048	—	32	128
D130	**16**	2048	1024	2048	256	1024	256	—	512	2048	**16**	2048	> 128	> 128
K033	2048	—	—	—	256	2048	256	—	2048	—	—	—	32	> 128
K126	—	—	—	—	—	—	1024	—	—	—	—	—	128	> 128
K130	2048	—	2048	—	1024	—	2048	—	2048	—	2048	—	64	> 128
2139	2048	—	2048	—	2048	—	2048	—	2048	—	256	—	> 128	> 128
K142	2048	—	2048	—	256	—	**32**	—	2048	—	128	—	32	> 128
K261	—	—	—	—	1024	—	256	—	2048	—	512	—	> 128	> 128
K272	2048	—	—	—	1024	2048	512	—	2048	—	—	—	32	> 128
K290	**32**	2048	2048	—	**128**	2048	256	1024	2048	—	512	—	> 128	> 128
K294	**64**	—	256	2048	256	—	512	2048	256	—	512	—	> 128	> 128
T076	512	2048	512	1024	512	512	**32**	128	512	1024	**128**	2048	> 128	> 128
T106	2048	—	1024	2048	—	—	1024	—	2048	—	—	—	16	> 128
T138	2048	—	2048	—	256	—	—	—	—	—	2048	—	128	> 128
T201	1024	—	1024	—	2048	—	2048	—	1024	—	2048	2048	> 128	> 128
PA124	512	—	1024	—	1024	—	256	2048	1024	—	2048	—	> 128	> 128

*Note:* /: MIC or MBC undetermined up to 2048 μg/mL; values in bold represent outstanding (MIC ≤ 32 μg/mL) and excellent activities (32 < MIC ≤ 128 μg/mL) for extracts.

Abbreviations: DMBE, dichloromethane/methanol bark extract; DMLE, dichloromethane/methanol leaf extract; HBE, hexane bark extract; HLE, hexane leaf extract; MBC, minimum bactericidal concentration; MBE, methanol bark extract; MIC, minimal inhibitory concentration; MLE, methanol leaf extract.

### 3.3. Effect of HLE on the Antioxidant System of *P. aeruginosa* D130

#### 3.3.1. Effect of HLE on Catalase Activity

**FIGURE 2:**
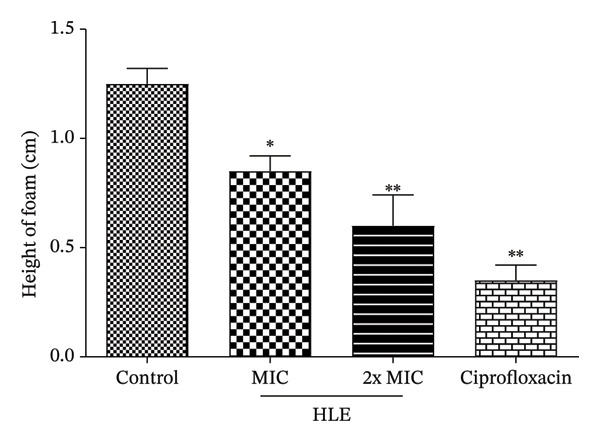
Effect of HLE on catalase activity of *P. aeruginosa* D130. MIC: minimum inhibitory concentration; HLE: hexane leaf extract. The MIC of ciprofloxacin and HLE against *P. aeruginosa* D130 was 16 μg/mL. Data are expressed as the mean ± SD; ^∗^(*p* < 0.05); ^∗∗^(*p* < 0.01).

In this investigation, we measured the effect of HLE on the catalase activity of *P. aeruginosa* D130. The findings suggest that HLE (MIC and 2xMIC) significantly (*p* < 0.05) decreased the catalase activity of *P. aeruginosa* D130. In contrast to HLE (0.60 ± 0.14 cm) and the control (1.25 ± 0.07 cm), ciprofloxacin (reference antibacterial) showed a more noticeable effect (0.35 ± 0.07 cm) (Figure 2).

#### 3.3.2. Effect of HLE on Lipid Peroxidation

**FIGURE 3:**
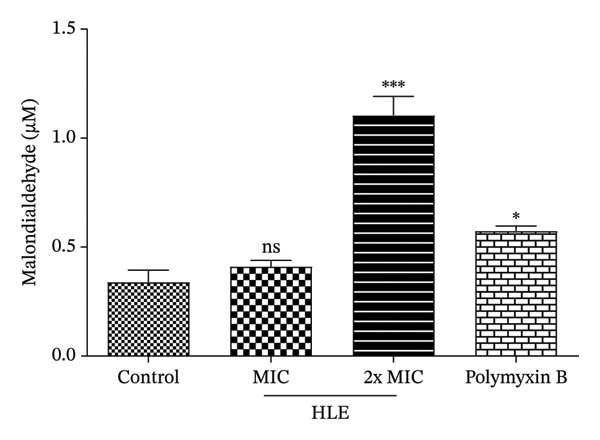
Effect of HLE on the malondialdehyde production in *P. aeruginosa* D130. MIC: minimum inhibitory concentration; HLE: hexane leaf extract. The MICs of polymyxin B and HLE against *P. aeruginosa* D130 were 32 and 16 μg/mL, respectively. Data are expressed as the mean ± SD; ns: not significant; ^∗^(*p* < 0.05); ^∗∗∗^(*p* < 0.001).

Exposure of *P. aeruginosa* D130 to HLE showed a significant (*p* < 0.05) and concentration‐dependent increase of MDA production, indicating that the tested extract induces lipid peroxidation in *P. aeruginosa* D130 (Figure 3). This was more pronounced at 2xMIC (1.11 ± 0.09 μM) compared to the control (0.34 ± 0.06 μM) and polymyxin B used as a reference antibacterial (0.57 ± 0.02 μM).

### 3.4. Enhancement of Antibiotic Activity

#### 3.4.1. Antibiotic Resistance Modulation Effect of Extracts

A preliminary test allowed the selection of the hexane, DMBE, and MBE of *A. gabonensis* at their subinhibitory concentration MIC/8 (Table S2, supporting information) to assess their antibiotic resistance modulation effect against selected clinical MDR *P. aeruginosa*. HBE enhanced the activity of amikacin on all isolates tested (100%), followed by that of gentamicin (83.33%), streptomycin (66.67%), and ciprofloxacin (50%) (Table 3). DMBE also enhanced the activity of amikacin on all isolates tested (100%), followed by gentamicin and cefixime (66.67%) (Table 4). On the other hand, MBE improved the activity of amikacin on all isolates tested (100%), followed by that of gentamicin and streptomycin (66.67%), and levofloxacin (50%) (Table 5). Overall, the most significant antibiotic resistance modulation effects of extracts were observed with aminoglycosides (amikacin, gentamicin, and streptomycin) regardless of the associated extract (Tables 3, 4, and 5).

**TABLE 3:** Antibiotic resistance modulation effect of HBE against MDR *P. aeruginosa*.

Antibiotics	Extract concentration	Bacteria, MIC (μg/mL), and modulation factor (in brackets)						AME (%)
		B026	K033	K126	K294	T201	PA124	
Ciprofloxacin	0 MIC/8	0.5 0.5 (1)	> 64 > 64 (nd)	16 1 (**16**)	16 8 (**2**)	2 4 (0.5)	4 ≤ 2 (> **2**)	50

Levofloxacin	0 MIC/8	16 32 (0.5)	32 ≤ 0.5 (> **64**)	16 16 (1)	16 ≤ 0.5	64 64 (1)	≤ 2 ≤ 2 (nd)	16.67

Streptomycin	0 MIC/8	128 128 (1)	32 16 (**2**)	128 64 (**2**)	256 64 (**4**)	256 256 (1)	256 ≤ 2 (> **128**)	66.67

Gentamicin	0 MIC/8	64 16 (**4**)	8 ≤ 2 (> **4**)	≤ 2 ≤ 2 (nd)	32 16 (**2**)	8 ≤ 2 (> **4**)	8 ≤ 2 (> **4**)	83.33

Amikacin	0 MIC/8	64 ≤ 2 (> **32**)	64 ≤ 2 (> **32**)	128 ≤ 2 (> **64**)	128 4 (**32**)	128 64 (**2**)	32 16 (**2**)	100

Cefixime	0 MIC/8	64 64 (1)	32 64 (0.5)	128 128 (1)	> 256 128 (≥ **2**)	128 128 (1)	256 128 (**2**)	33.33

Cefotaxime	0 MIC/8	256 256 (1)	256 64 (**4**)	16 16 (1)	> 256 > 256 (nd)	64 128 (0.5)	128 256 (0.5)	16.67

*Note:* AME: antibiotic resistance modulation effect; values in bold represent modulation factors ≥ 2.

Abbreviations: MIC, minimum inhibitory concentration; nd, not determined.

**TABLE 4:** Antibiotic resistance modulation effect of DMBE against MDR *P. aeruginosa*.

Antibiotics	Extract concentration	Bacteria, MIC (μg/mL), and modulation factor (in brackets)						AME (%)
		B026	K033	K126	K294	T201	PA124	
Ciprofloxacin	0 MIC/8	0.5 ≤ 0.5 (≥ 1)	> 64 > 64 (nd)	16 ≤ 0.5 (> **32**)	16 16 (1)	2 2 (1)	4 ≤ 2 (> **2**)	33.33

Levofloxacin	0 MIC/8	16 2 (**8**)	32 ≤ 0.5 (> **64**)	16 16 (1)	16 8 (**2**)	64 > 64 (≤ 1)	≤ 2 ≤ 2 (nd)	50

Streptomycin	0 MIC/8	128 128 (1)	32 64 (0.5)	128 64 (**2**)	256 256 (1)	256 > 256 (≤ 1)	256 64 (**4**)	33.33

Gentamicin	0 MIC/8	64 64 (1)	8 ≤ 2 (> **4**)	≤ 2 ≤ 2 (nd)	32 ≤ 2 (> **16**)	8 ≤ 2 (> **4**)	8 ≤ 2 (> **4**)	66.67

Amikacin	0 MIC/8	64 4 (**16**)	64 8 (**8**)	128 8 (**16**)	128 8 (**16**)	128 4 (**32**)	32 ≤ 2 (> **16**)	100

Cefixime	0 MIC/8	64 32 (**2**)	32 4 (**8**)	128 128 (1)	> 256 128 (> **2**)	128 128 (1)	256 64 (**4**)	66.67

Cefotaxime	0 MIC/8	256 256 (1)	256 64 (**4**)	16 64 (0.25)	> 256 > 256 (1)	64 128 (0.5)	128 64 (**2**)	33.33

*Note:* AME: antibiotic resistance modulation effect; values in bold represent modulation factors ≥ 2.

Abbreviations: MIC: minimum inhibitory concentration; nd: not determined.

**TABLE 5:** Antibiotic resistance modulation effect of MBE against MDR *P. aeruginosa*.

Antibiotics	Extract concentration	Bacteria, MIC (μg/mL), and modulation factor (in brackets)						AME (%)
		B026	K033	K126	K294	T201	PA124	
Ciprofloxacin	0 MIC/8	0.5 32 (0.015)	> 64 > 64 (nd)	16 ≤ 0.5 (> **32**)	16 32 (0.5)	2 4 (0.5)	4 ≤ 2 (> **2**)	33.33

Levofloxacin	0 MIC/8	16 32 (0.5)	32 ≤ 0.5 (> **64**)	16 16 (1)	16 ≤ 0.5 (> **32**)	64 4 (**16**)	≤ 2 ≤ 2 (nd)	50

Streptomycin	0 MIC/8	128 128 (1)	32 32 (1)	128 64 (**2**)	256 256 (1)	256 ≤ 2 (> **128**)	256 128 (> **2**)	66.67

Gentamicin	0 MIC/8	64 32 (**2**)	8 256 (0.03)	≤ 2 ≤ 2 (nd)	32 ≤ 2 (> **16**)	8 ≤ 2 (> **4**)	8 ≤ 2 (> **4**)	66.67

Amikacin	0 MIC/8	64 4 (**16**)	64 8 (**8**)	128 16 (**8**)	128 ≤ 0.5 (> **256**)	128 ≤ 2 (> **64**)	32 ≤ 2 (> **2**)	100

Cefixime	0 MIC/8	64 > 256 (≤ 0.25)	32 256 (0.125)	128 128 (1)	> 256 128 (> **2**)	128 128 (1)	256 128 (> **2**)	33.33

Cefotaxime	0 MIC/8	256 256 (1)	256 64 (**64**)	16 16 (1)	> 256 > 256 (nd)	64 128 (0.5)	128 128 (1)	16.67

*Note:* AME: Antibiotic resistance modulation effect; values in bold represent modulation factors ≥ 2.

Abbreviations: MIC, minimum inhibitory concentration; nd, not determined.

#### 3.4.2. Interaction Effects Between Antibiotics and *A. gabonensis* Extracts Against Selected MDR *P. aeruginosa*

**FIGURE 4:**
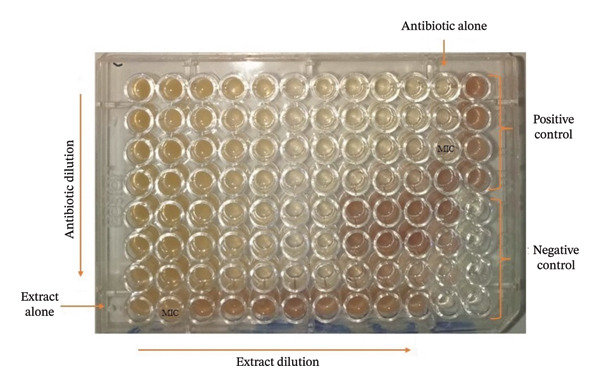
96‐well microplate illustrating a result of a checkerboard assay.

The interactions between extracts (MBE and HBE) and aminoglycosides (amikacin, gentamicin, and streptomycin) were evaluated through the determination of their fractional inhibitory concentration index (∑FIC) using the checkerboard assay. Figure 4 presents a 96‐well microplate illustrating the result of the checkerboard assay. The results showed that these extracts in combination with antibiotics exhibited synergy (∑FIC ≤ 0.5), particularly with amikacin and streptomycin against the tested isolates (Tables 6 and 7). However, cases of additive effect (0.5 ≤ ∑FIC ≤ 1) were observed with the combination of HBE and gentamicin or amikacin against *P. aeruginosa* PA124 (Table 6); MBE with gentamicin against *P. aeruginosa* T201; and MBE with streptomycin against *P. aeruginosa* PA124 (Table 7). Furthermore, indifference (1 ≤ ∑FIC ≤ 2) was observed with gentamicin and streptomycin in association with HBE on *P. aeruginosa* PA124 (Table 6).

**TABLE 6:** Interaction effects between antibiotics and the HBE against selected MDR *P. aeruginosa*.

Antibiotics	Bacteria	Fractional inhibitory concentration		∑FIC	Interactions
		HBE	ATB		
Streptomycin	K294	0.063	0.008	0.071	Synergy
	T201	0.008	0.125	0.133	Synergy
	PA124	0.135	1	1.135	Indifference

Gentamicin	K294	0.031	0.031	0.062	Synergy
	T201	0.5	0.125	0.625	Additivity
	PA124	1	0.125	1.125	Indifference

Amikacin	K294	0.031	0.031	0.062	Synergy
	T201	0.031	0.031	0.062	Synergy
	PA124	0.016	0.5	0.516	Additivity

*Note:* ATB: antibiotic; ƩFIC: Fractional Inhibitory Concentration index.

Abbreviation: HBE: hexane bark extract.

**TABLE 7:** Interaction effects between antibiotics and the MBE against selected MDR *P. aeruginosa*.

Antibiotics	Bacteria	Fractional inhibitory concentration		∑FIC	Interaction
		MBE	ATB		
Streptomycin	K294	0.062	0.008	0.07	Synergy
	T201	0.016	0.25	0.266	Synergy
	PA124	0.031	0.5	0.531	Additivity

Gentamicin	K294	0.008	0.031	0.039	Synergy
	T201	0.5	0.125	0.625	Additivity
	PA124	0.031	0.25	0.281	Synergy

Amikacin	K294	0.031	0.031	0.062	Synergy
	T201	0.004	0.031	0.035	Synergy
	PA124	0.25	0.031s	0.281	Synergy

*Note:* ATB: antibiotic; ƩFIC: Fractional Inhibitory Concentration index.

Abbreviation: MBE: methanol bark extract.

## 4. Discussion

MDR bacteria pose a threat to human health and a growing challenge for medicine [43]. Among these bacteria, MDR *P. aeruginosa* has been considered a serious threat due to its high‐level intrinsic resistance [2, 5]. It is therefore necessary to find new treatment alternatives against bacterial infections. Many reports have demonstrated the evidence of plants as important sources of new substances with the potential to fight antimicrobial resistance alone or in combination with conventional antibiotics against many bacteria, including MDR *P. aeruginosa* [8–10, 44]. In light of this, this study aimed to evaluate the antibacterial activity of *Allanblackia gabonensis* extract alone and in combination with antibiotics against MDR *P. aeruginosa.* Additionally, a preliminary phytochemical composition of the extracts was carried out.

Regarding the study of the antibacterial activity, the methanol and hexane extracts of the bark and leaves of *A. gabonensis* showed the best activities, with exceptional (MIC ≤ 32 μg/mL) and excellent (32 < MIC ≤ 128 μg/mL) activities on at least one of the *P. aeruginosa* isolates tested compared to other extracts. The variability in activity from one extract to another may be due to the difference in extraction solvents used, but also to the parts of the plant used. In fact, the affinity of various plant metabolites to common extraction solvents has been established, and their presence and quantity may vary accordingly [45]. Similarly, these metabolites are not always distributed in equal proportions in the plant organs. Thus, the observed activity would be due to the presence of secondary metabolites detected in the tested extracts, which are known for their antibacterial properties [46]. The results obtained are in agreement with the work of Fankam et al., who reported antibacterial activity of methanol extracts from different organs of *A. gabonensis* against Gram‐negative bacteria with MICs ranging from 16 to 1024 μg/mL on 75% of the isolates tested [24]. Similarly, Nganou et al. showed that some compounds, such as morelloflavone and guttiferone BL, isolated from *A. gabonensis*, exhibited antibacterial activity with MICs ranging from 8 to 512 μg/mL on Gram‐negative bacteria [23]. As far as we know, the antibacterial activity of the HLE and HBE extracts of *A. gabonensis* is presented herein for the first time, indicating their potential as complementary resources for identifying new antipseudomonal agents.

Faced with the resurgence of resistant bacteria, the discovery of new antibacterial drugs requires the exploration of new targets with modes of action different from those of conventional antibiotics. In this study, we evaluated the effect of one of the most active extracts of *A. gabonensis* (HLE) on the antioxidant system of *P. aeruginosa* D130, particularly on catalase and lipid peroxidation. Previous investigations have indicated that mutant pathogens lacking catalase exhibit increased vulnerability to oxidative stress and host immune system attacks [37, 47]. In this study, HLE inhibited the catalase activity of *P. aeruginosa* D130. In addition, the extracts caused lipid peroxidation of *P. aeruginosa* D130. It is known that catalase generated under oxidative stress conditions can reduce lipid peroxidation by decreasing reactive oxygen species (ROS), thus leading to decreased lipid peroxidation [48]. Indeed, ROS are known to chemically react with proteins, lipids, and other cellular biomolecules, thereby causing alterations and oxidative modifications that damage bacterial cellular activities [49]. Likely, secondary metabolites such as phenolic compounds, triterpenes, and alkaloids detected in this extract are responsible for this inhibition. For example, phenols are known to inhibit microorganism growth through iron deprivation or hydrogen bonding with vital proteins, disrupting the synthesis of proteins essential for bacterial life [50]. Although some studies have already demonstrated the antimicrobial properties of *A. gabonensis*, this work presents for the first time the ability of HLE to inhibit the bacterial antioxidant system, making it a potential source for antipseudomonal drugs.

Restoring the activity of antibiotics by combining them with natural substances appears as a strategy to effectively combat resistance in bacteria. In fact, it has been shown that the plant ingredients combined with antibiotics may improve their activity or facilitate their interaction with their target inside the bacteria and thus prevent the emergence of resistance [9–11, 39]. Synergistic interaction of the natural substances with existing antibiotics effectively addresses the issue of resistance. It not only enhances the sensitivity of MDR bacteria but also decreases the toxicity of some antibiotics [10]. In this study, we evaluated the combination effect of *A. gabonensis* extracts with seven antibiotics to improve their activities against MDR *P. aeruginosa*. At their subinhibitory concentration (MIC/8), these extracts potentiated 2‐ to 256‐fold the activity of the tested antibiotics, particularly that of aminoglycosides. Moreover, the HBE and MBE exhibited synergy (∑FIC ≤ 0.5) in combination with antibiotics, particularly with aminoglycosides (amikacin, gentamicin, and streptomycin) against the tested isolates. These improvements in the activity of the tested antibiotics suggest, on the one hand, that the constituents present in these extracts could act as adjuvants to antibiotics by inhibiting resistance mechanisms, including the expression of efflux pumps and the main resistance mechanism in *P. aeruginosa* [2, 51]. Moreover, previous studies have shown that resistance to aminoglycosides in MDR *P. aeruginosa* is significantly associated with the expression of efflux pumps [52]. Based on that, the tested extracts probably modulate the antibiotic resistance by interfering with efflux pumps in the test organisms. In fact, many phytochemicals are known for their efflux pump inhibitory potential. For instance, a steroidal alkaloid known as conessine was shown to inhibit the MexAB‐OprM efflux pump in *P. aeruginosa* and restore the antibiotic activity [53]. Previous studies have demonstrated that plant extracts and their phytochemicals, as well as their nanoparticles, can act in synergy with commonly used antibiotics against microorganisms, including drug‐resistant *P. aeruginosa* [16, 54, 55]. The observed synergy could be explained by the fact that the antibiotic and the extract act on different bacterial targets [11]. Since aminoglycosides act by inhibiting protein synthesis through binding to the ribosomal subunits [56], it can be assumed that the tested extracts act on the bacterial membrane, thus facilitating the entry and action of the antibiotics.

## 5. Conclusions

Overall, this study brings new evidence regarding the antimicrobial properties of *A. gabonensis* as potential candidates for the discovery of new antibiotics, as well as resistance modulators to fight infections caused by MDR *P. aeruginosa*. The HLE and HBE of *A. gabonensis* may serve as candidates for the discovery of new antibiotics, while its bark extracts could be used in combination with antibiotics to manage infections involving MDR *P. aeruginosa.* The future research on this plant can be shifted to the isolation and identification of its active ingredients. Moreover, the evaluation of the toxicity and the *in vivo* activity of the most active extracts should be envisaged. This can bring important contributions to the development of new molecules useful against infections due to MDR *P. aeruginosa*.

**Nomenclature**
ATCC: American Type Culture Collection
DMSO: Dimethyl‐sulfoxide
DMBE: Dichloromethane/methanol bark extract
DMLE: Dichloromethane/methanol leaf extract
FIC: Fractional inhibitory concentration
GAE: Gallic acid equivalents
HBE: Hexane bark extract
HLE: Hexane leaf extract
INT: Iodonitrotetrazolium chloride
MBC: Minimum bactericidal concentration
MBE: Methanol bark extract
MDA: Malondialdehyde
MDR: Multidrug‐resistant
MHA: Mueller–Hinton agar
MHB: Mueller–Hinton broth
MIC: Minimum inhibitory concentration
MLE: Methanol leaf extract
ROS: Reactive oxygen species
TPC: Total phenolic content

## Author Contributions

Céline Brinda Sonfack: investigation, data curation, writing–original draft, and writing–review and editing. Aimé Gabriel Fankam: conceptualization, methodology, resources, supervision, validation, data curation, formal analysis, writing–original draft, and writing–review and editing. Brenda Ngueffo Tiwa: investigation and writing–review and editing. Michael Francis Kengne: resources and review and editing. Armelle Tsafack Mbaveng: resources and review and editing; Victor Kuete: resources and review and editing.

## Funding

The authors received no specific funding for this study.

## Disclosure

All authors read and approved the final manuscript.

## Conflicts of Interest

The authors declare no conflicts of interest.

## Supporting Information

Table S1. Bacteria used and their resistance features.

Table S2. Preliminary results for the antibiotic resistance modulation acitivity of *A. gabonensis* extracts against PA124.

## Supporting information


**Supporting Information** Additional supporting information can be found online in the Supporting Information section.

## Data Availability

The data that support the findings of this study are available from the corresponding author upon reasonable request.
