# Supplementary material for: Phytochemical Composition and Antipseudomonal Activity of Allanblackia gabonensis (Clusiaceae) Extracts Alone and With Antibiotics Against Drug‐Resistant Clinical Isolates
Source: Scientifica (Cairo). 2026 Mar 2;2026:6070077. doi: 10.1155/sci5/6070077 (PMC12951695; doi:10.1155/sci5/6070077)
Supplement: Supplementary file 1 — Supporting Information Additional supporting information can be found online in the Supporting Information section. [file SCI5-2026-6070077-s001.pdf]

Table S1. Bacteria used and their resistance features

Bacteria	Features	Reference
ATCC27853	Reference strain	American type culture collection
B026	MDR clinical isolate : IMP^{r}, AMX^{r}, CAZ^{r}, FOX^{r}, CTX^{r}, CXM^{r}, COT^{r}, CIP^{r}, FOX^{r}, NAL, COL^{r}, PRL^{r}, PPT^{r}, TCC^{r}, TET^{r}, NIT^{r}, AMC^{r}	
B178	MDR clinical isolate : AMX^{r}, CAZ^{r}, FOX^{r}, CXM^{r}, CIP^{r}, FOX^{r}, NAL^{r}, COL^{r}, PRL^{r}, PPT^{r}, TCC^{r}, TET^{r}, NIT^{r}, AMC^{r}.	
D100	MDR clinical isolate : CTX^{r}, CXM^{r}, COT^{r}, AMK^{r}, GEN^{r}, COL^{r}, PRL^{r}, TCC^{r}, TET^{r}, NIT^{r}, AMC$^{r.}$	
D130	MDR clinical isolate : IMP^{r}, AMX^{r}, CAZ^{r}, FOX^{r}, CTX^{r}, CXM^{r}, COT^{r}, NAL, COL, PRL, PPT, TCC, TET, NIT, AMC	
K033	MDR clinical isolate : IMP, AMX, CAZ, FOX, CTX, CXM, COT, CIP, OFX, NAL, COL, PRL, PPT, TCC, TET, NIT, AMC.	
K126	MDR clinical isolate : IMP, AMX, CAZ, FOX, CTX, CXM, COT, AMK, CIP, O FX, NAL, COL, PRL, PPT, TCC, TET, NIT, AMC	
K130	MDR clinical isolate : IMP, AMX, CAZ, FOX, CTX, CXM, COT, CIP, OFX, NAL, COL, PRL, PPT, TCC, TET, NIT, AMC	
K139	MDR clinical isolate : IMP, CAZ, FOX, CTX, CXM, COT, CIP, OFX, NAL, COL, PRL, PPT, TCC, TET, NIT, AMC	
K142	MDR clinical isolate : IMP, AMX, CAZ, FOX, CTX, CXM, COT, CIP, OFX, NAL, COL, PRL, PPT, TCC, TET, NIT, AMC	[1]
K261	MDR clinical isolate : AMX, CAZ, FOX, CTX, CXM, COT, CIP, OFX, NAL, COL, PRL, PPT, TCC, TET, NIT, AMC	
K272	MDR clinical isolate : IMP, AMX, CAZ, FOX, CTX, CXM, COT, CIP, OFX, NAL, COL, PRL, PPT, TCC, TET, NIT, AMC	
K290	MDR clinical isolate : AMX, CAZ, FOX, CTX, CXM, COT, NAL, COL, PRL, PPT, TCC, TET, NIT, AMC.	
K294	MDR clinical isolate : IMP, AMX, CAZ, FOX, CTX, CXM, COT, CIP, OFX, NAL, COL, PRL, PPT, TCC, TET, NIT, AMC.	
TO76	MDR clinical isolate : IMP, AMX, CAZ, FOX, CXM, COT, CIP, OFX, NAL, COL, PRL, PPT, TCC, TET, NIT, AMC.	
T106	MDR clinical isolate : IMP, AMX, CAZ, FOX, CTX, CXM, COT, CIP, OFX, NAL, COL, PRL, PPT, TCC, TET, NIT, AMC	
T138	MDR clinical isolate : IMP, AMX, CAZ, FOX, CTX, CXM, COT, CIP, OFX, NAL, COL, PRL, PPT, TCC, TET, NIT, AMC.	
T201	MDR clinical isolate : IMP, AMX, CAZ, FOX, CTX, CXM, AMK, GEN, COT, CIP, OFX, NAL, PRL, PPT, TCC, TET, NIT, AMC	
PA124	MDR clinical isolate	[2]

FOX : cefoxitin, AMC : amoxicillin-clavulanic acid, IMI : imipenem, AMI : amikacin, GEN : gentamicin, CIP : ciprofloxacin, OFX : ofloxacin, CO : trimethoprim-sulfamethoxazole, TET : tetracycline, NIT: nitrofurantoin, NAL: nalixidic acid, CTX : cefotaxime, CXM : cefuroxime, CAZ : ceftazidime, PRL : piperacillin, PPT: piperacillin-tazobactam, TTC: ticarcillin-clavulanic acid, r: resistant.

References

[1] Kengne MF, Mbaveng AT, Karimo O, Dadjo BST, Tsobeng OD, Marbou WJT, Kuete V. Frequency of Fecal Carriage of ESBL Resistance Genes in Multidrug-Resistant *Pseudomonas*

aeruginosa Isolates from Cancer Patients at Laquintinie Hospital, Douala, Littoral Region, Cameroon. Int J Microbiol. 2024 Jun 11;2024:7685878. https://doi.org/10.1155/2024/7685878.
[2] Lorenzi V, Muselli A, Bernardini AF, Berti L, Pagès JM, Amaral L, Bolla JM. Geraniol restores antibiotic activities against multidrug-resistant isolates from gram-negative species. Antimicrob Agents Chemother. 2009 May;53(5):2209-11. https://doi.org/10.1128/AAC.00919-08.

Table S2. Preliminary results for antibiotic resistance modulation *A. ganbonensis* extracts against PA124

Antibiotics	Extract concentration	Extracts, MIC (µg/mL), and modulation factor (in brackets) [a]					
		**HLE**	**DMLE**	**MLE**	**HBE**	**DMBE**	**MBE**
Ciprofloxacin	0	4	4	4	4	4	4
	CMI/8	≤2 (>**2**)	≤2 (>**2**)	≤2 (>**2**)	≤2 (>**2**)	≤2 (>**2**)	≤2 (>**2**)
Levofloxacin	0	≤2	≤2	≤2	≤2	≤2	≤2
	CMI/8	≤2 (nd)	≤2 (nd)	≤2 (nd)	≤2 (nd)	≤2 (nd)	≤2 (nd)
Streptomycin	0	256	256	256	256	256	256
	CMI/8	256 (1)	256 (1)	256 (1)	≤2 (**>128**)	64 (**4**)	128 (**>2**)
Gentamycin	0	8	8	8	8	8	8
	CMI/8	8 (1)	8 (1)	8 (1)	≤2 (>**4**)	≤2 (>**4**)	≤2 (>**4**)
Amikacin	0	32	32	32	32	32	32
	CMI/8	32 (1)	8 (**4**)	8 (4)	16 (**2**)	≤2 (>**16**)	≤2 (>**2**)
Cefixime	0	256	256	256	256	256	256
	CMI/8	128 (**2**)	256 (1)	128 (**2**)	128 (**2**)	64 (**4**)	128 (**>2**)
Cefotaxime	0	128	128	128	128	128	128
	CMI/8	256 (0,5)	128 (1)	64 (**2**)	256 (0,5)	64 (**2**)	128 (>1)

[a] Values in bolds represent modulation factors ≥ 2.

HLE: hexane leaf extract; DMLE: dichloromethane/methanol leaf extract; MLE: methanol leaf extract; HBE: hexane bark extract; DMBE: dichloromethane/methanol bark extract; MBE: methanol bark extract;
